# Comparative analysis of the liver transcriptome in the red-eared slider (*Trachemys scripta elegans*) post exposure to noise

**DOI:** 10.1371/journal.pone.0305858

**Published:** 2024-08-01

**Authors:** Guangwei Ma, Ziye Zuo, Handong Li, Xiaofei Zhai, Tongliang Wang, Jichao Wang

**Affiliations:** Ministry of Education Key Laboratory for Ecology of Tropical Islands, Key Laboratory of Tropical Animal and Plant Ecology of Hainan Province, College of Life Sciences, Hainan Normal University, Haikou, China; University of Bari Aldo Moro: Universita degli Studi di Bari Aldo Moro, ITALY

## Abstract

Exposure to noise can cause non-auditory health problems and has been widely studied in mammals such as rats and rabbits. However, the non-auditory effects of noise exposure on reptiles (such as red-eared sliders) remain unclear. In this study, we determined the noise exposure-induced transcriptomic changes in the liver of red-eared slider (*Trachemys scripta elegans*) using Illumina Novaseq6000 sequencing technology. The transcriptome analysis identified 176 differentially expressed genes (DEGs), which were mainly enriched in lipid metabolism. KEGG analysis showed that by affecting the peroxisome proliferator activated receptor (PPAR) signaling pathway these DEGs increased lipid synthesis and decreased lipid oxidation. The Oil Red O staining results validated our data that noise exposure increased hepatic lipid deposition. Thus, noise exposure may lead to lipid accumulation and toxicity, mitochondrial damage, and accelerated oxidative stress. Our findings provide insights into the molecular process underlying non-auditory damage caused by noise exposure in *T*. *scripta elegans*.

## Introduction

Noise is defined as "the unwanted, unpleasant or disagreeable sound that causes discomfort to all living beings" [1]. Generally, a whispering sound is measured around 15 decibels (dB), normal conversations around 60 dB, a jet engine can measure close to 120 dB, and above 85 dB is considered harmful [1]. Exposure to noise can cause several health problems, which are generally divided into auditory (such as noise-induced hidden hearing loss) and non-auditory (such as changes in the metabolic pathways in liver) effects [2]. The non-auditory health effects of noise have been widely studied in mammals [2–4]. Previous studies have shown that exposure to high noise levels causes hepatotoxicity in rats [5, 6]. Hematoxylin and eosin staining experiments indicated significant swelling and lipidosis in the liver tissue of New Zealand white rabbits post exposure to 100 dB white noise for 8 h per day for two weeks [7]. However, most studies on the effects of noise exposure in reptiles have focused on the auditory system [8], while harm to the non-auditory system has rarely been reported.

Noise exposure can cause oxidative stress in animals, and the liver is an important organ with a potent antioxidant mechanism to counterbalance the effects of oxidants and relieve environmental stress [9]. Several genes have been reported to be associated with the maintenance of the oxidation-reduction balance in the liver. Jin et al. have reported that *FOXO1b*, *Bax*, *Caspase9*, and *c-Myc* genes play important roles in the response to oxidative stress induced by high concentrations of ammonia in the liver of *Ctenopharyngodon idellus* [10]. Hong et al. have described that the red-eared slider (*Trachemys scripta elegans*) responds to salinity-induced oxidative stress by up-regulation of the expression of *KCNH5*, *STK32*, and *SIK1* in the liver to balance the entry of sodium and chloride ions [11]. Noise exposure can also cause oxidative stress in animals. Zhang et al. reported the response mechanism of hybrid sturgeon to oxidative stress caused by continuously played ship noise, which 588 genes were differentially expressed in the liver, involved in various life activities such as DNA replication, apoptosis, and lipid metabolism [12].

Turtles are ancient reptiles, also known as living fossils, with fossil records dating back over 200 million years [13]. They form an evolutionarily unique and morphologically distinct vertebrate clade. Red-eared sliders (*T*. *scripta elegans*) are freshwater turtles that are remarkably adaptable to the environment and are listed as one of the 100 most threatening aliens worldwide by the International Union for Conservation of Nature. Previous studies have shown that *T*. *scripta elegans* has stronger anti-oxidative stress ability than native species in response to environmental stress [14]. Therefore, *T*. *scripta elegans* is the default animal model of turtle used in environmental stress studies. To understand the intrinsic molecular processes of the *T*. *scripta elegans* liver during noise exposure, we determined the transcriptome changes in the liver using Illumina Novaseq6000 sequencing technology. The findings of the present study are expected to provide insights into the molecular processes underlying non-auditory damage caused by noise exposure in *T*. *scripta elegans*.

## Materials and methods

### Animals and ethics statement

Similar to other exposure experiments [11], six 2-year-old healthy *T*. *scripta elegans* juvenile males (BW: 418–430 g) were purchased from farms in Hainan Province, China (110.7191700° E, 19.8033100° N). They were acclimated to a cement pool half-filled with freshwater for two weeks. Turtles were fed commercial diets on Tuesday and Friday, and after 24 h of feeding, unused feed was siphoned out and one-third of the water in the pool was replaced. After acclimatization, all turtles were randomly allocated to two groups: (1) noise group, exposed to 115 dB sound pressure level (SPL) white nose binaurally for 24 h; and (2) control group, exposed to 25 dB SPL white noses binaurally for 24 h. All animal treatment procedures were approved by the Animal Research Ethics Committee of the Hainan Provincial Education Centre for Ecology and Environment, Hainan Normal University (HNECEE-2022-006). All efforts were aimed at minimizing animal suffering and the number of turtles sacrificed.

### Noise exposure

The experiments were conducted in a soundproof room, and the turtles were kept unrestrained in plastic boxes (80×40×30 cm^3^; one turtle per box). There was a small amount of water in the box and the water was just submerged the ventral armor, in which case the turtle could not sink its head into the water to avoid the noise in air. The loudspeaker (A66, Ying Huang Audio Equipment Co., LTD., Guangzhou, China) was placed 90 cm above the bottom of the plastic box, and continuously played the noise for 24 h. The SPLs changed by no more than 2 dB across the box. The turtles of Noise Group or Control Group were exposed to 115 or 25 dB SPL white noses binaurally for 24 h, respectively. The noise exposure experiment was repeated three times, and during noise exposure, all the turtles were fasted and kept under the same conditions, differing only in noise exposure.

### Sample collection, RNA extraction, and Illumina sequencing

After 24 h of noise exposure, six turtles were sampled on the same day, and general anesthesia was administered. Tricaine (MS-222) was injected as an anesthetic into the hind limb muscles. 30 minutes later, the turtles were stimulated with tweezers on the hind limbs and observed. If there was no response, it meant that they had entered deep anesthesia; conversely, if there was a significant response, then the injection was continued according to the 20% of the previous dosage, until they entered deep anesthesia. All turtles were finally moved to -20°C for 0.5–1 h for euthanasia [11]. The liver of each turtle was sampled, and stored at -80°C until used for RNA extraction. Total RNA was extracted from each liver sample using a TRIzol kit (Invitrogen, Carlsbad, CA, USA) following the manufacturer’s instructions. The RNA quality was assessed using an Agilent 2100 Bioanalyzer (Agilent Technologies, Palo Alto, CA, USA) and verified using RNase-free agarose gel electrophoresis. After total RNA extraction, the eukaryotic mRNA was enriched with oligo (dT) beads. Enriched mRNA was fragmented into short fragments using fragmentation buffer and reverse-transcribed into cDNA using the NEB Next Ultra RNA Library Prep Kit (NEB#7530; New England Biolabs, Ipswich, MA, USA). Purified double-stranded cDNA fragments were end-repaired, A-tailed, and ligated to Illumina sequencing adapters. The ligation reaction was purified using AMPure XP Beads (1.0X). The ligated fragments were subjected to size selection using agarose gel electrophoresis and polymerase chain reaction (PCR). The resulting cDNA library was sequenced using Illumina Novaseq6000 by Gene Denovo Biotechnology Co. (Guangzhou, China), and 150 bp paired-end reads were generated.

### Transcriptomic analyses

Reads obtained from sequencing machines include raw reads containing adapters or low-quality bases that affect subsequent assembly and analysis. Clean reads were obtained using fastp (version 0.18.0) [15]. Bowtie2 (version 2.2.8) was used to map the reads to ribosome RNA (rRNA) database [16]. Paired-end clean reads were assembled by mapping to the reference genome (accession number GCA_013100865.1) [17] using HISAT2 [18]. Transcriptome data were submitted to the Genome Sequence Archive (GSA, https://bigd.big.ac.cn/gsa/) under the accession number CRA010451. Differentially expressed genes (DEGs) were identified using edgeR (R package) [19] based on a threshold false discovery rate (FDR) < 0.05, and an absolute fold change (FC) ≥ 2 (|log2 (FC)| > 1). The enrichment analyses for DEGs were performed based on the Gene Ontology (GO) [20] and Kyoto Encyclopedia of Genes and Genomes (KEGG) [21] databases using KOBAS 3.0 software [22]. The *P*-value was adjusted using the Benjamini Hochberg false discovery rate, and terms with a corrected *P*-value (Q value) < 0.05 were recognized as significant for GO and KEGG enrichment analysis.

### Oil Red O staining

To assess fat deposition in the liver, the Oil Red O Staining was performed. After 24 h of noise exposure and euthanasia, the liver of each turtle was sampled and fixed in 4% paraformaldehyde at room temperature for 24 h. Then, the liver tissue was embedded in the optimal cutting temperature (OCT) compound and 10 μm thick sections were created using a cryostat (Leica Biosystems, Wetzlar, Germany). Cryostat sections were stained with Oil Red O (in 70% isopropyl alcohol) and observed under a microscope.

## Results

### Transcriptome assembly and annotation

Six cDNA libraries were constructed in this study, and 50.52 Gb of raw data were obtained from the liver transcriptomes of six *T*. *scripta elegans* individuals (S1 Table). By trimming sequencing adapters/poly-N and removing poor-quality reads, 50.09 Gb of clean reads was obtained for downstream analysis (S1 Table). Bowtie2 was used for removing the reads which were mapping to the ribosome, and the unmapped reads were retained for subsequent transcriptome analysis (S2 Table). Hisat2 was used for the reference genome-based alignment analysis, and the mapping rates of the six samples ranged from 85.44% to 86.78% (S3 Table and S1 Fig).

### Identification of DEGs

**Fig 1:**
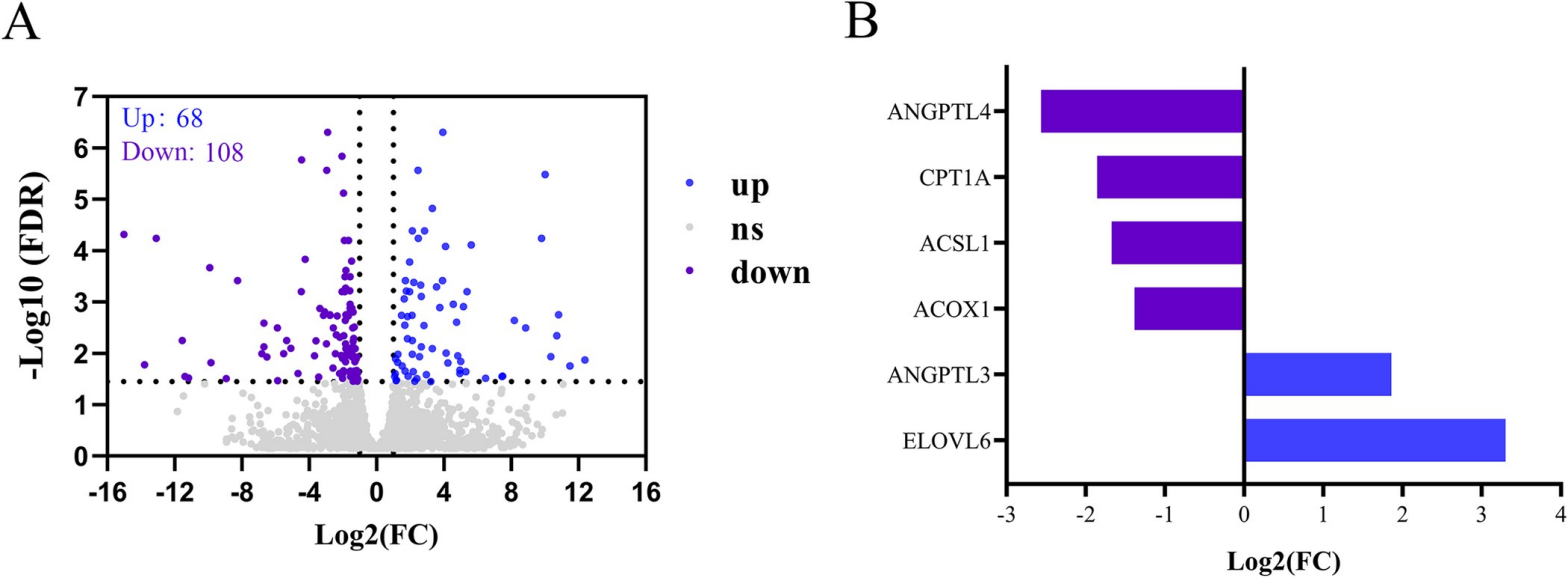
Differentially-expressed genes (DEGs) identified in the liver of *T*. *scripta elegans* after noise exposure. **A:** Blue dots indicate up-regulated genes; purple dots indicate down-regulated genes; **B:** Blue columns indicate up-regulated genes, and purple columns indicate down-regulated genes.

To reveal the transcriptome changes of *T*. *scripta elegans* liver under noise exposure, we performed differential gene expression analysis (based on the criteria of two-fold or greater change and FDR < 0.05) in the control group relative to the noise group. In total, 176 genes (68 up-regulated and 108 down-regulated) were identified as significant DEGs in the liver tissues of *T*. *scripta elegans* between the control and noise groups (Fig 1A and S4 Table). Fig 1B shows the six featured DEGs in the liver tissue of the control and noise-treated groups.

### Gene ontology enrichment analysis of DEGs

**Fig 2:**
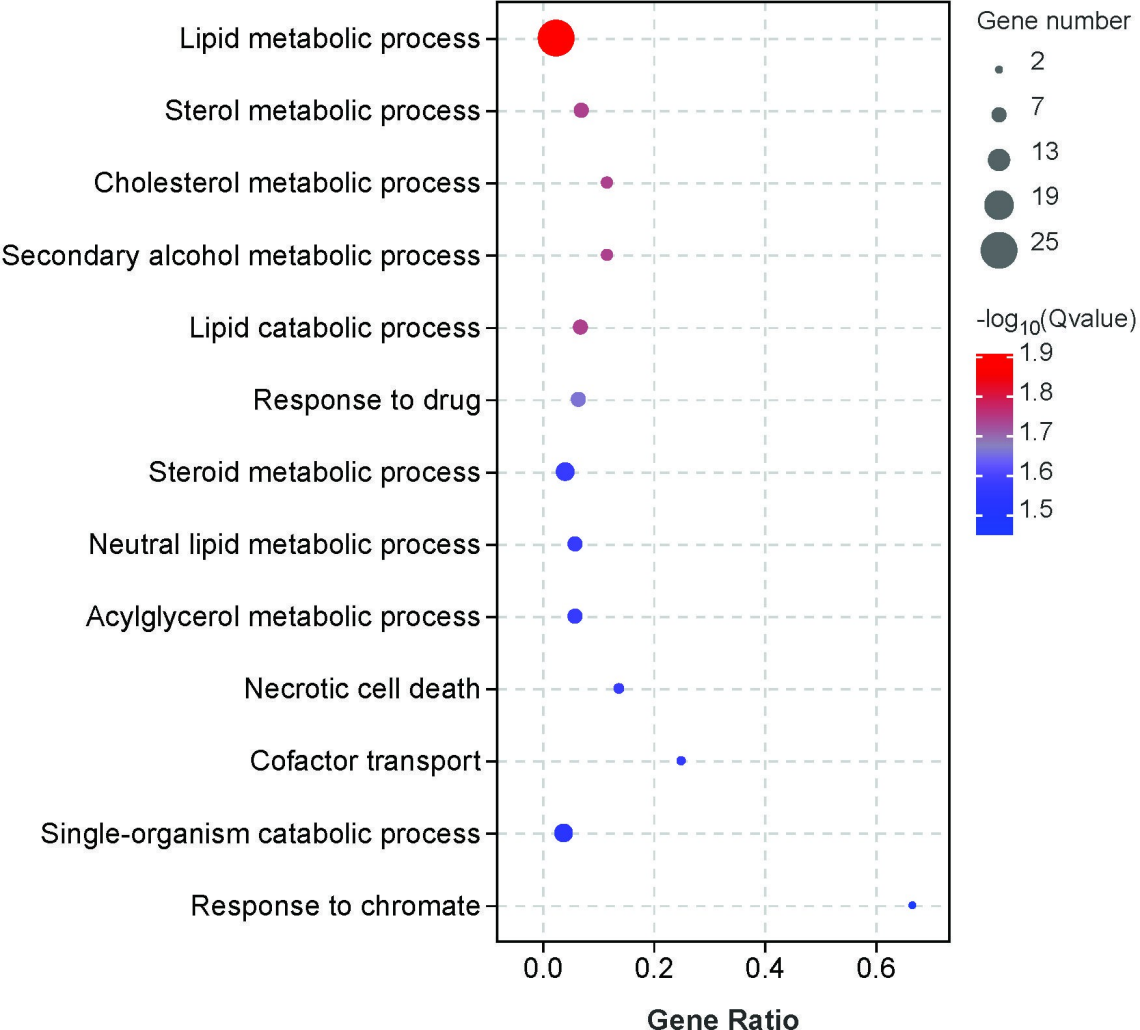
Significant GO terms enriched based on the DEGs in control vs. noise groups.

To gain insight into the biological roles of these DEGs, we performed a GO category enrichment analyses. GO enrichment data indicated that these DEGs were classified into two major functional categories: Biological Processes (BPs) and Cellular Components (CCs) (Fig 2 and S5 Table). In the GO BP category, the DEGs were mainly enriched in lipid metabolic processes (GO:0006629), lipid catabolic processes (GO:0016042), neutral lipid metabolic processes (GO:0006638), acylglycerol metabolic processes (GO:0006639), and single-organism catabolic processes (GO:0044712). These terms are closely associated with lipid synthesis and decomposition. Other highlighted BPs included sterol metabolic processes (GO:0016125), cholesterol metabolic processes (GO:0008203), and steroid metabolic processes (GO:0008202) related to sterol synthesis and decomposition. For the GO CC category, the DEGs were mainly enriched in primary lysosome (GO:0005766), extracellular region part (GO:0044421), extracellular region (GO:0005576), sex chromosome (GO:0000803) and germ cell nucleus (GO:0043073). See S5 Table for the specific significance and other information on these terms.

### KEGG enrichment analysis of DEGs

**Fig 3:**
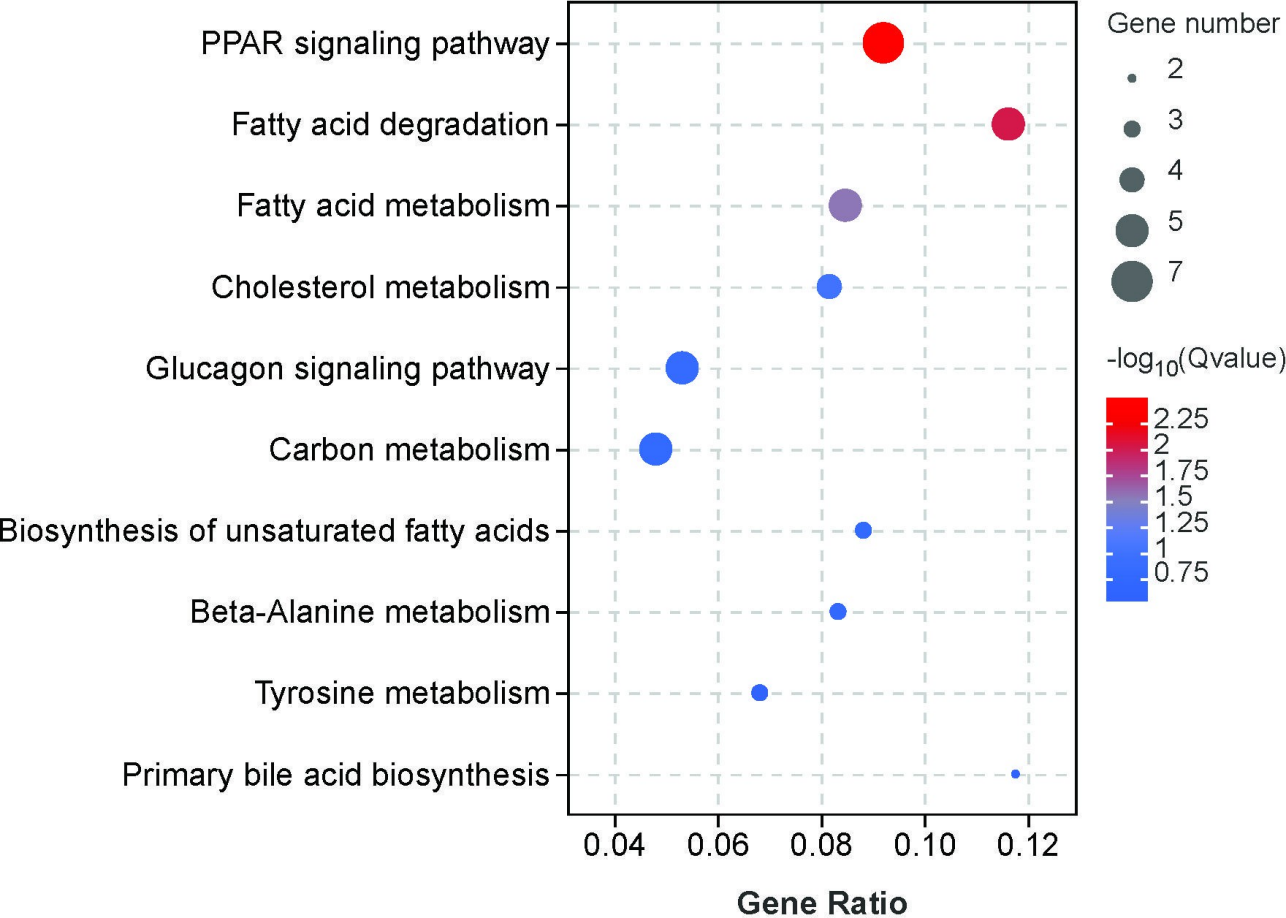
Top 10 KEGG pathway enrichment associated with the DEGs in control vs. noise groups.

The results of KEGG analysis showed that these DEGs were successfully annotated and assigned to 205 pathways. The significant KEGG pathways were peroxisome proliferator activated receptor (PPAR) signaling pathway (ko03320), fatty acid degradation (ko00071), and fatty acid metabolism (ko01212) pathways (S6 Table). For clarity and concise presentation, Fig 3 shows the top 10 altered pathways.

### Noise exposure increased lipid droplet accumulation

**Fig 4:**
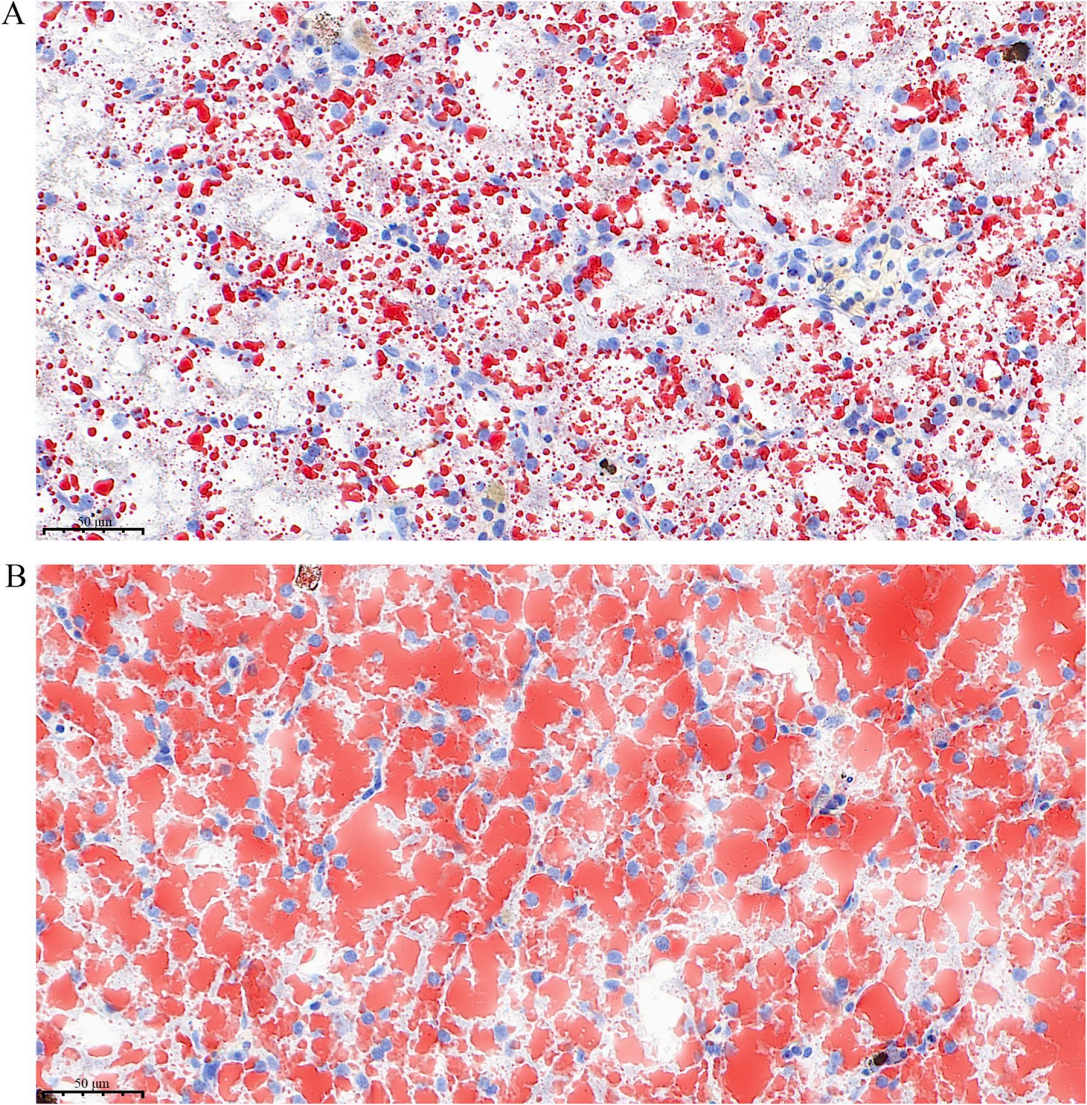
Representative OIL RED O stained frozen liver section. **A:** Oil Red O staining of liver tissue from the control group; **B:** Oil Red O staining of liver tissue from the noise group.

Based on the transcriptome results, we hypothesized that noise exposure induced hepatic lipid deposition. We evaluated hepatic steatosis via Oil Red O staining. The Oil Red O stained lipid droplets were dramatically higher in the noise group than those in the control group (Fig 4).

## Discussion

Noise exposure is harmful to both humans and animals. In our study, *T*. *scripta elegans* were exposed to 115 dB SPL white noise, and RNA-seq technology was used to build the gene expression profile of the liver. Transcriptome sequencing results showed that noise caused significant changes in the expression of 176 genes in the liver tissue of *T*. *scripta elegans*. These DEGs increased lipid synthesis and decreased lipid oxidation, leading to lipid accumulation, which causes lipid toxicity, induces mitochondrial damage, and accelerates oxidative stress by regulating the PPAR signaling pathway.

Environmental stress can affect the metabolism and immune system of turtles. Changes in the metabolic pathways in the liver are important for turtles to adapt to adverse environments. For example, *T*. *scripta elegans* can adapt to salinity stress by balancing the entry of sodium and chloride ions and accumulating plasma urea and free amino acids by altering gene expression in the liver tissue [11]. Another study showed that the DEGs in the liver that were enriched in NF-κB signaling pathway played certain protective roles in Chinese Strip-necked Turtle (*Mauremys sinensis*) under ammonia exposure [23]. Recent research has shown 17 up-regulated and 13 down-regulated miRNAs to play important roles in the down-regulation of central metabolic processes and maintenance of tissue homeostasis in response to freezing stress in the Midland painted turtle (*Chrysemys picta marginata*) liver tissue [24]. In the present study, we focused on the effect of noise stress on turtles and found that noise caused significant changes in the expression of 176 genes in the livers of *T*. *scripta elegans*.

Previous studies have mainly focused on the effects of noise on the transcriptome of the auditory system, such as the cochlea [25, 26]. Few studies have been conducted on the transcriptome of the non-auditory system. Zhang et al. reported 588 DEGs in the livers of hybrid sturgeons and inhibition of lipid metabolism under simulated ship engine noise stress (exposure: 12 h) [12]. Similar to this previous study, the identified DEGs caused by noise exposure in our study were mainly enriched in lipid metabolic processes, lipid catabolic processes, neutral lipid metabolic processes, and acylglycerol metabolic processes. These DEGs included *ACSL1*, *ANGPTL3*, and *ELOVL6* (Figs 1B and 2 and S5 Table), suggesting the possibility of a lipid metabolism disorder in the liver of exposed *T*. *scripta elegans*.

Long-chain acyl-CoA synthetase-1 (ACSL1) is a key enzyme in the oxidative metabolism of fatty acids in the mitochondria [27]. In palmitic acid-treated human proximal tubular epithelial cells, the expression of NF-E2-related factor 2 (Nrf2) is inhibited, resulting in decreased expression of *ACSL1* gene, which leads to increased lipid deposition, and subsequently, increased ROS production and oxidative stress [28]. Angiopoietin like protein 3 (ANGPTL3) is a lipoprotein lipase (LPL) inhibitor that is primarily expressed and secreted by hepatocytes. A previous study has demonstrated that the siRNA-mediated knockdown of liver *ANGPTL3* relieved its inhibitory effect on LPL and significantly reduced hypertriglyceridemia and oxidative stress in nephrotic rats [29]. ELOVL family member 6 (ELOVL6) is a microsomal enzyme that regulates the elongation of C12-16 saturated and monounsaturated fatty acids. Studies in mice have shown that ELOVL6 promotes high-fat diet-induced liver oxidative stress [30], and that the absence of ELOVL6 decreases hepatic lipid accumulation and oxidative stress in low-density lipoprotein receptor-deficient mice [31]. In summary, noise exposure may lead to hepatic lipid accumulation, cause lipid metabolism disturbances in the liver of *T*. *scripta elegans*, and accelerate oxidative stress by up-regulating the expression of *ANGPTL3* and *ELOVL6* and down-regulating the expression of *ACSL1*.

Lipid accumulation in the liver is caused by a combination of reduced fatty acid oxidation and increased fat synthesis [32]. The PPAR signaling pathway is closely related to lipid metabolism and is a lipid sensor that modulates whole-body energy metabolism [33]. PPARs have three isoforms, PPARα, PPARβ and PPARγ. PPARα promotes fatty acid oxidation and transport by regulating genes involved in lipoprotein metabolism In contrast, PPARγ is a master regulator of adipogenesis that increases triglyceride formation and storage in lipid droplets. PPARβ stimulates lipid and glucose utilization by increasing mitochondrial function and fatty acid desaturation pathways [34]. In our study, the DEGs caused by noise exposure were mainly enriched in the PPAR signaling pathway, including *CPT1A*, *ACOX1*, *ANGPTL4*, and *ACSL1* (Figs 1B and 3 and S6 Table), suggesting the possibility of abnormal lipid accumulation in *T*. *scripta elegans* liver. Our Oil Red O staining results validated our speculation that noise exposure increased hepatic lipid deposition (Fig 4).

Carnitine palmitoyltransferase-1A (CPT1A) is the rate-limiting enzyme involved in long-chain fatty acid β-oxidation. In ulcerative colitis mice, down-regulation of CPT1A inhibits PPARα signaling pathway [35]. Acyl-CoA oxidase 1 (ACOX1) is the first rate-limiting enzyme involved in fatty acid oxidation and is regulated by PPARα [36, 37]. Angiopoietin-like protein 4 (ANGPTL4) is a lipoprotein lipase inhibitor that is a target gene of PPARα in the liver tissue [38, 39]. Silencing of PPARα aggravates lipid deposition and decreases the expression of *ANGPTL4* in non-alcoholic fatty liver disease cells [40]. In addition, the knockdown of *ACSL1* increases the expression of PPARγ in human liver cells [41]. Based on the above studies, we hypothesize that noise exposure decreased the expression of *ACSL1*, which up-regulated the expression of PPARγ and increased lipid synthesis. Furthermore, reduction of *CPT1A* expression inhibited the PPARα activity, which in turns reduced the expression of PPARα downstream target genes, *ACOX1* and *ANGPTL4*, thereby suppressing lipid oxidation. Increased lipid synthesis and decreased lipid oxidation leads to lipid accumulation, which causes lipid toxicity, induces mitochondrial damage, and accelerates oxidative stress.

Sexual dimorphism is prevalent in turtles, for example, sexually dimorphic hearing sensitivity has evolved in *T*. *scripta elegans* [42]. Further comparative transcriptomics analysis results showed that six DEGs (*GABRA1*, *GABRG2*, *GABBR2*, *GNAO1*, *SLC38A1*, and *SLC12A5*) in the GABAergic synapse pathway were identified to explain the differences in sexually dimorphic hearing sensitivity [43]. In our study, our experimental animals were six 2-year-old healthy *T*. *scripta elegans* males. It is worth investigating whether there are different molecular mechanisms in female *T*. *scripta elegans* to response to noise exposure in the future.

## Conclusion

In this study, we report the first transcriptome analysis of *T*. *scripta elegans* exposed to noise. We identified a total of 176 DEGs, which were mainly enriched in lipid metabolism. KEGG pathway analysis showed that by affecting the PPAR signaling pathway, these DEGs increased lipid synthesis and decreased lipid oxidation, leading to lipid accumulation, lipid toxicity, induced mitochondrial damage, and accelerated oxidative stress. The results of this study provide clues regarding the molecular process of damage caused by noise exposure.

## Supporting information

S1 Fig Mapping rate of 6 samples to the reference genome. (TIF)

S1 Table Data filtering statistics. (XLSX)

S2 Table Comparison of ribosome statistics. (XLSX)

S3 Table Summary of transcriptome assembly mapping to the genome. (XLSX)

S4 Table Summary of differential expression of genes. (XLSX)

S5 Table Significant GO terms enriched based on the DEGs in control vs. noise groups. (XLSX)

S6 Table Significant KEGG pathways enriched based on the DEGs in control vs. noise groups. (XLSX)
