# Supplementary figures and images for: Comparative analysis of the liver transcriptome in the red-eared slider (Trachemys scripta elegans) post exposure to noise
Source: PLoS One. 2024 Aug 1;19(8):e0305858. doi: 10.1371/journal.pone.0305858 (PMC11293744; doi:10.1371/journal.pone.0305858)

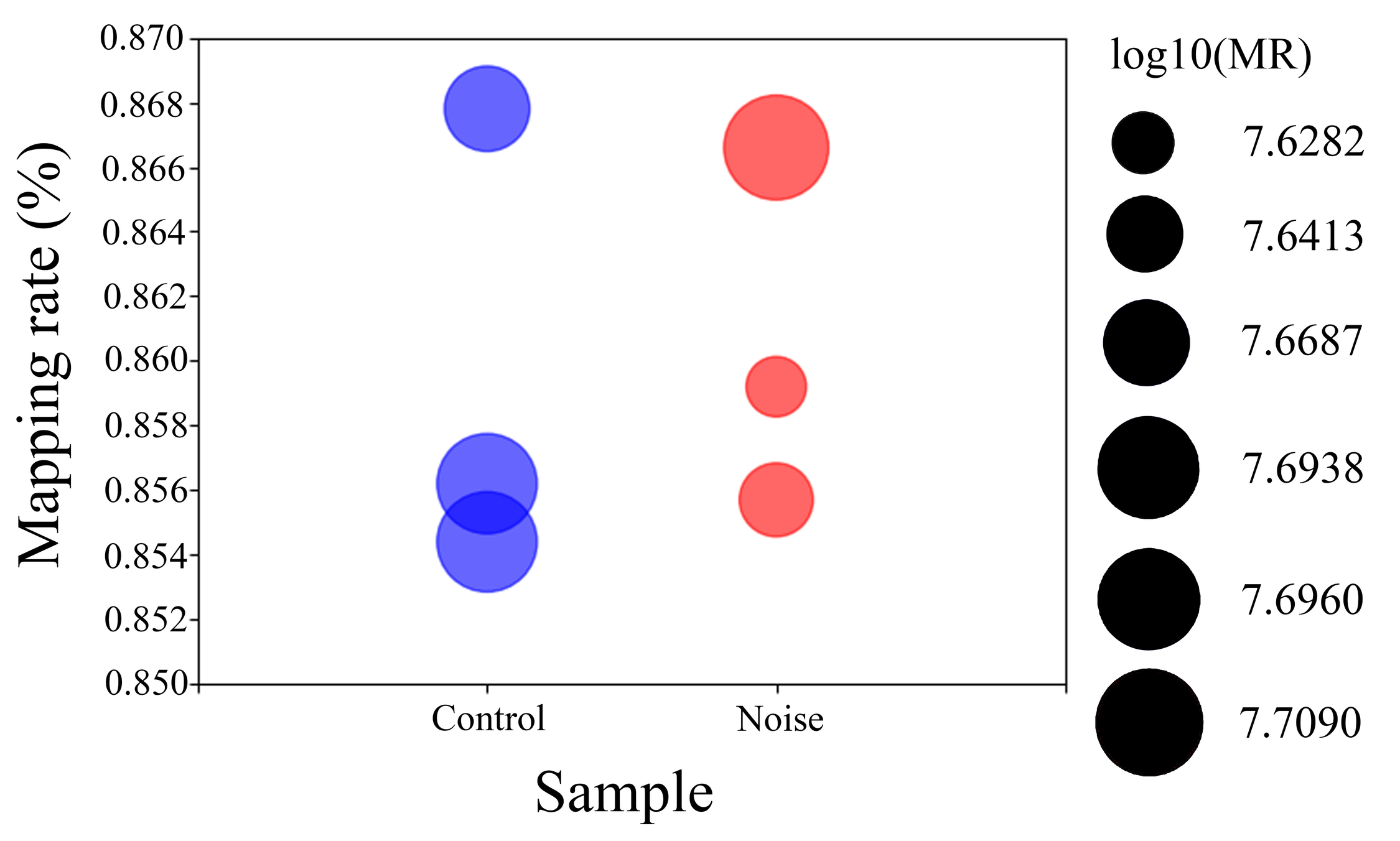

Supplement: S1 Fig — (TIF) [file pone.0305858.s001.tif]
